# Germline Variants in DNA Damage Repair Genes and *HOXB13* Among Black Patients With Early-Onset Prostate Cancer

**DOI:** 10.1200/PO.22.00460

**Published:** 2022-11-29

**Authors:** Matthew R. Trendowski, Christopher Sample, Tara Baird, Azita Sadeghpour, David Moon, Julie J. Ruterbusch, Jennifer L. Beebe-Dimmer, Kathleen A. Cooney

**Affiliations:** ^1^ Department of Oncology, Wayne State University School of Medicine, Detroit, MI; ^2^ Department of Medicine, Duke University School of Medicine, Durham, NC; ^3^ Barbara Ann Karmanos Cancer Institute, Detroit, MI; ^4^ Duke Cancer Institute, Durham, NC

## Abstract

Genetic studies of prostate cancer susceptibility have predominantly focused on non-Hispanic White men, despite the observation that Black men are more likely to develop prostate cancer and die from the disease. Therefore, we sought to identify genetic variants in Black patients diagnosed with early-onset prostate cancer.

**METHODS:**

Whole-exome sequencing of germline DNA from a population-based cohort of Black men diagnosed with prostate cancer at age 62 years or younger was performed. Analysis was focused on a panel of DNA damage repair (DDR) genes and *HOXB13*. All discovered variants were ranked according to their pathogenic potential based upon REVEL score, evidence from existing literature, and prevalence in the cohort. Logistic regression was used to investigate associations between mutation status and relevant clinical characteristics.

**RESULTS:**

Among 743 Black prostate cancer patients, we identified 26 unique pathogenic (P) or likely pathogenic (LP) variants in 14 genes (including *HOXB13*, *BRCA1/2*, *BRIP1*, *ATM*, *CHEK2*, and *PALB2*) among 30 men, or approximately 4.0% of the patient population. We also identified 33 unique variants of unknown significance in 16 genes among 39 men. Because of the rarity of these variants in the population, most associations between clinical characteristics did not achieve statistical significance. However, our results suggest that carriers for P or LP (P/LP) variants were more likely to have a first-degree relative diagnosed with DDR gene-associated cancer, have a higher prostate-specific antigen at time of diagnosis, and be diagnosed with metastatic disease.

**CONCLUSION:**

Variants in DDR genes and *HOXB13* may be important cancer risk factors for Black men diagnosed with early-onset prostate cancer, and are more frequently observed in men with a family history of cancer.

## INTRODUCTION

Advances in prostate cancer detection and therapy have enabled patients to live for years beyond their initial diagnoses, as the average 5-year relative survival rate for all stages is nearly 100% and the average 15-year survival rate is nearly 95%.^1^ Nevertheless, prostate cancer is still the most frequently diagnosed cancer in adult men in the United States (248,530 cases per year), and ranks second in overall cancer mortality for this patient population (34,130 deaths per year).^1^ Black men are disproportionately affected by prostate cancer, with the highest incidence rate of any US population (202 per 100,000 persons) and are more than twice as likely to die from the disease compared with non-Hispanic White men. Powell et al^2^ demonstrated that Black men diagnosed with early-onset prostate cancer have higher age-specific mortality rates. Men initially diagnosed with stage IV disease have a much poorer prognosis, and the incidence of metastatic disease at presentation has increased slightly over the past two decades, from 4% in 2003 to 6% in 2021.^3^ Black men are also more likely to have metastatic disease at diagnosis.^4^

**CONTEXT**

**Key Objective**
Although it has been demonstrated that Black men are at increased susceptibility to develop prostate cancer and die from the disease, studies examining genetic predisposition have been limited. To address this health disparities gap, we sought to identify genetic variants in Black men diagnosed with early-onset prostate cancer.
**Knowledge Generated**
Germline variants in DNA damage repair (DDR) genes and *HOXB13* found to be pathogenic or likely pathogenic were identified in this patient cohort. Carriers of these variants often had a family history of cancer, and were associated with both high-risk disease and an increased susceptibility to developing prostate cancer at an earlier age.
**Relevance**
Genetic variation in DNA damage repair genes and *HOXB13* may be an important risk factor for Black men diagnosed with early-onset prostate cancer. Further investigation of these genetic associations will provide insight into the unique susceptibility Black men have to developing prostate cancer, potentially reducing current health disparities.

The explanation for the poorer outcomes among Black men diagnosed with prostate cancer is not well understood, but includes both biologic and nonbiologic causes. Differences in access to health care contribute to the disparity; a recent large study of men receiving care in the Veterans Affairs health system with equal access to care showed that Black men in this system did not present with more aggressive and/or metastatic disease and had similar outcomes compared with non-Hispanic White men.^5^ Furthermore, relatively few risk factors are firmly established for prostate cancer and none appear to account for these racial differences. There is evidence that inherited genetic susceptibility accounts for up to 40% of all prostate cancer cases, even more so among individuals diagnosed with early-onset prostate cancer.^6-8^ Considerable effort has been taken to examine the potential influence of germline genetic variation on prostate cancer susceptibility and its potential contributions to racial disparities in incidence and mortality. These investigations have been hampered by lower participation of Black men in genetic research studies, as both prostate cancer association and linkage studies have disproportionately examined patients of European ancestry.^9^

Despite the fact that prostate cancer has strong evidence of heritability, it has been very challenging to identify rare prostate cancer susceptibility genes that contribute significantly to prostate cancer incidence. Our research team was successful in using linkage analysis and candidate gene sequencing to identify *HOXB13* as a prostate cancer susceptibility gene.^10^ A recurrent nonsynonymous change was identified, which results in the nonconservative substitution of glutamic acid for glycine (G84E), in probands from four unrelated prostate cancer families. This variant was shown to be more prevalent in men with early-onset prostate cancer and/or a positive family history of prostate cancer compared with noncarriers. Further research uncovered that the variant occurs on a common haplotype consistent with a founder allele and is almost exclusively seen in White men.^11^ More recently, multiple studies have confirmed the important role of variants in DNA repair genes in prostate cancer susceptibility and aggressiveness.^12,13^

We and others have shown that men with early-onset prostate cancer are more likely to harbor both rare and common genetic variants associated with prostate cancer.^6,10,14^ In a previous study of 96 Black prostate cancer survivors, we identified three protein truncating variants in both *BRCA2* and *BRIP1* associated with early-onset disease (≤ 55 years at diagnosis), demonstrating that rare variants may contribute to disease onset in this patient population.^15^ In the current study, we seek to expand upon these findings by extensively characterizing the spectrum of rare germline genetic variants in a cohort of Black men diagnosed with early-onset prostate cancer. Specifically, we focused on 35 known cancer susceptibility genes (Table 1), primarily in DNA damage repair (DDR) pathways. In addition, we determined whether identified risk alleles are associated with epidemiologic and clinical characteristics that are relevant to prostate cancer prognosis.

**TABLE 1.**
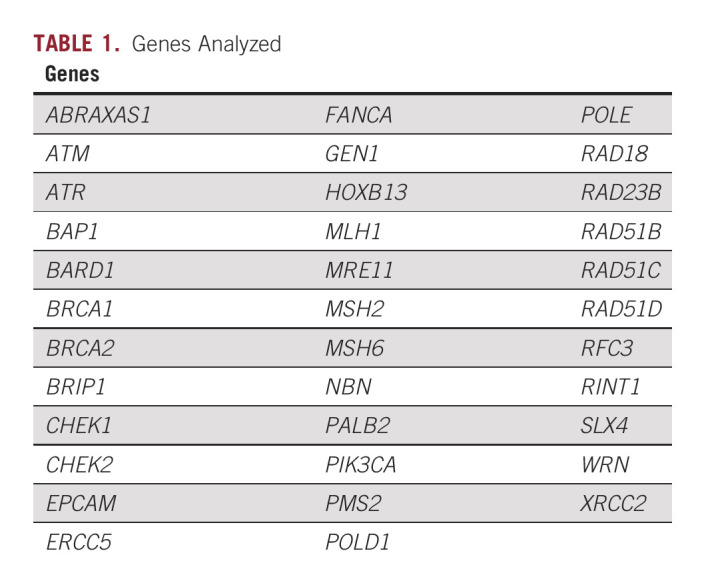
Genes Analyzed

## METHODS

### Study Population

The Early-onset Prostate Cancer cohort is a unique, population-based cohort of Black men younger than or 62 years diagnosed with prostate cancer. Eligible prostate cancer cases were identified from the Metropolitan Detroit Cancer Surveillance System (MDCSS) cancer registry. In addition, clinical data on all consented participants were abstracted from MDCSS; including date and age at diagnosis, biopsy and prostatectomy Gleason grade, tumor stage, and first course of treatment. All participants completed a survey to collect key demographic information, medical history, physical activity, tobacco and alcohol use, family history of cancer (breast, ovarian, prostate, kidney, pancreatic, colorectal, lung, liver, and other), screening practices, prostate cancer treatment(s), and any evidence of disease recurrence. Subjects were asked to provide a blood or saliva sample for genetic studies. DNA extraction was performed according to standard protocols. The research protocols and study documentation were approved by institutional review boards of the respective institutions and USAMRDC Human Research Protection Office. Informed consent was obtained from all patients.

### DNA Sequencing and Variant Annotation

Extracted genomic DNA was prepared for whole-exome sequencing, and sequencing was performed at the Sequencing and Genomic Technologies Shared Resource, part of the Duke University School of Medicine (Durham, NC). A custom pipeline on the basis of GATK best practices was used for variant calling.^16^ All pathogenic (P) and likely pathogenic (LP) variants were confirmed using Sanger sequencing. Filtered variants and multiple-nucleotide variants (Data Supplement) were individually assessed for classification into three categories: (1) benign or likely benign (B/LB), (2) variant of uncertain significance (VUS), or (3) pathogenic or likely pathogenic (P/LP). VarSome (releases 9-10)^17^ was used to establish base American College of Medical Genetics (ACMG) classification criteria,^18^ which were then adjusted to align with Sequence Variant Interpretation-Working Group (SVI-WG) recommendations.^19^ Three researchers (A.S., C.S., and D.M.) reviewed all variants independently and, if needed, formed a consensus for conflicting classifications. Further details on sample inclusion, sequencing technology, and data processing are provided in the Data Supplement.

### Statistical Analysis

The distributions for demographic and clinical characteristics were described using counts and percentages, as were the P/LP variants and VUS identified in this study population. Prostate-specific antigen (PSA) was dichotomized as a binary variable using < 10 ng/mL as the cutoff value. Gleason score at the time of radical prostatectomy was taken over Gleason score at biopsy and categorized as 7 (3 + 4) or less versus 7 (4 + 3) or higher. Three separate variables were created on the basis of reported family history of cancer: (1) first-degree family history of prostate cancer, (2) first-degree family history of any cancer, and (3) first-degree family history of DDR gene–associated cancers. DDR gene–associated cancers were defined as breast, ovarian, prostate, kidney, and pancreatic cancers. Logistic regression was used to estimate the odds and 95% CIs for having a P/LP variant for each of the clinical characteristics, excluding patients with a VUS. Separate analyses examined carrier status of a VUS (excluding patients who had a P/LP variant) and P/LP DDR gene carriers. All analyses were performed in R 3.3.2,^20^ and an alpha of 0.05 was set to determine statistical significance.

## RESULTS

### Cohort Characteristics

The median age at diagnosis among the 743 Black participants was 56 years (range, 38-62 years), and 256 (34.5%) of men were diagnosed before age 55 years (Table 2). Nearly 30% of men had a positive family history of prostate cancer in at least one first-degree relative, while approximately 60% of men had a first-degree relative diagnosed with any type of cancer. Unfavorable intermediate- to high-risk prostate cancer (defined as having a Gleason score of 4 + 3 and higher at diagnosis, tumor stage T3 and higher, or any T stage with evidence of lymph node involvement or metastatic disease) was identified in 294 (40%) of men. This included 150 men (20.2%) and 26 men (3.5%) who presented with evidence of regional or distant stage disease, respectively.

**TABLE 2.**
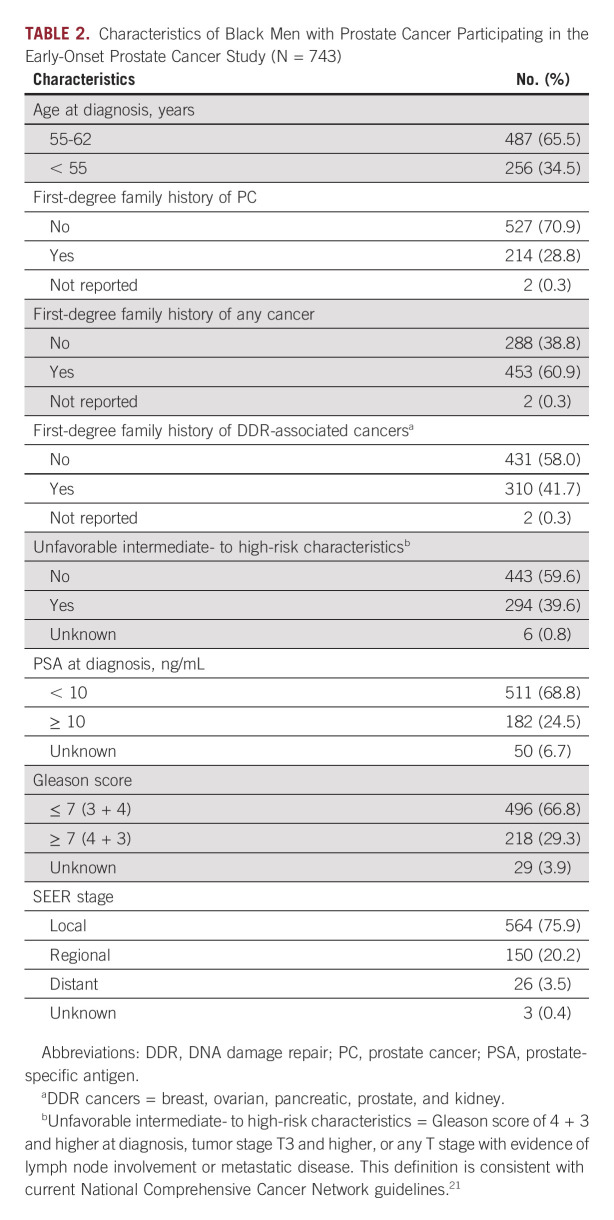
Characteristics of Black Men with Prostate Cancer Participating in the Early-Onset Prostate Cancer Study (N = 743)

### Analysis of Germline Variants

**FIG 1.**
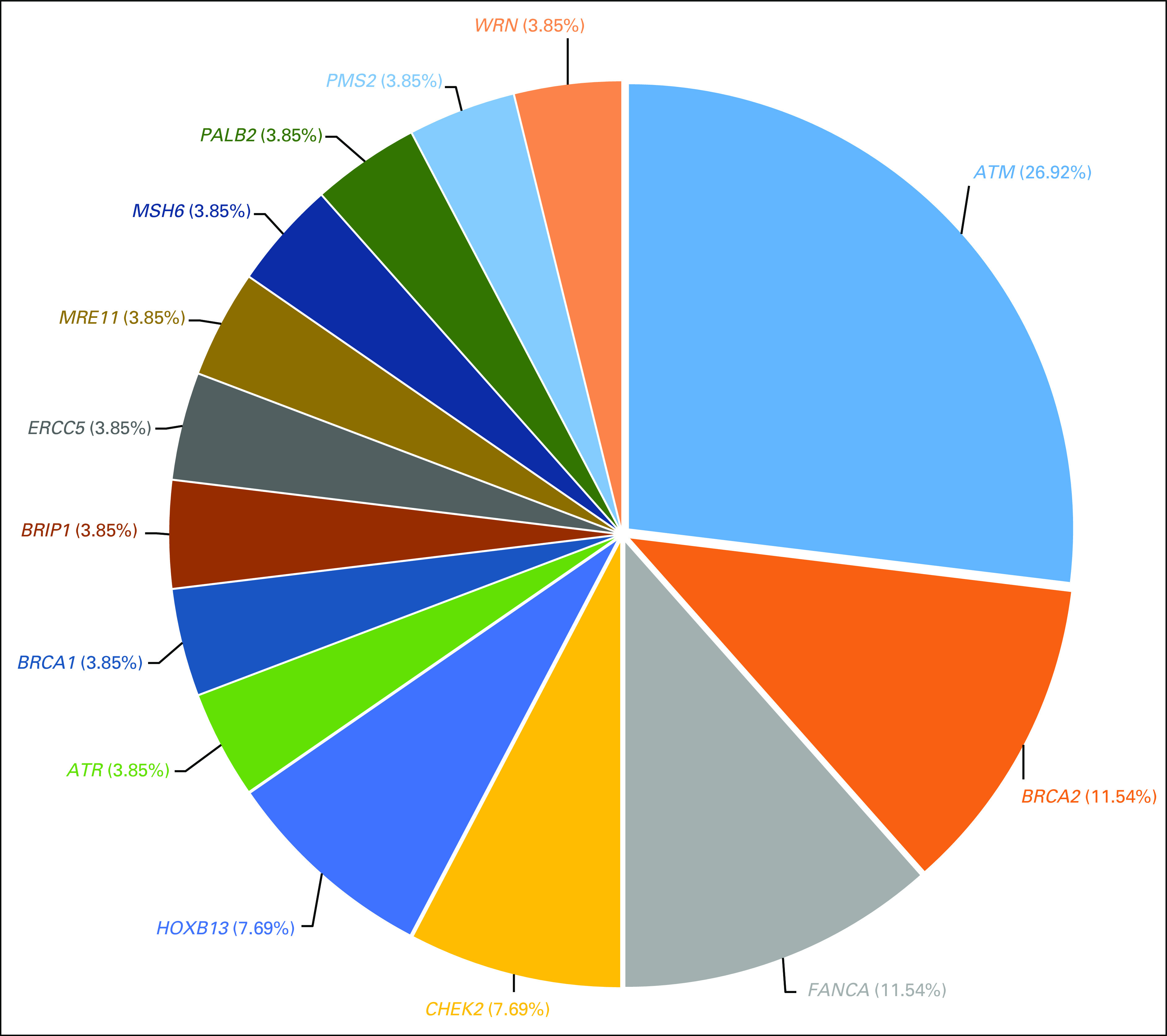
Frequency of identified unique pathogenic/likely pathogenic germline variants in genes.

In this cohort of Black men diagnosed with early-onset prostate cancer (≤ 62 years), we discovered 26 rare P/LP variants in 14 genes among 30 men, or approximately 4.0% of the patient population (Table 3 and Fig 1). We observed P/LP variants in *HOXB13* and the following DDR genes: *ATM*, *ATR*, *BRCA1*, *BRCA2*, *BRIP1*, *CHEK2*, *ERCC5*, *FANCA*, *MRE11*, *MSH6*, *PALB2*, *PMS2*, and *WRN*. One participant had two variants in *ATM* (p.Asn81LysfsTer19 and p.Val2716Ala). All variants were seen in only one person with the exception of two variants in *HOXB13* (G84E [Gly84Glu] and X285K [Ter285LysfsTer97]) each observed in three patients, and one variant in *ATR* (Ile774AsnfsTer3) observed in two patients.

**TABLE 3.**
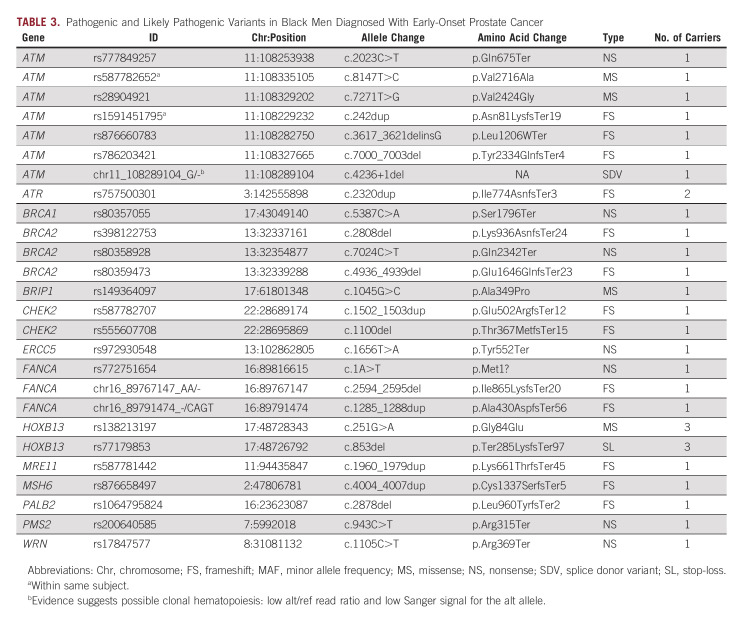
Pathogenic and Likely Pathogenic Variants in Black Men Diagnosed With Early-Onset Prostate Cancer

We also identified 33 unique VUS that were associated with our patient cohort in 16 genes among 39 men (Data Supplement). As opposed to the P/LP variants, many of the VUS were found in multiple individuals. Of note, *PMS2* was highly represented among these patients, as four individuals had a *PMS2* p.Leu166Pro variant and two individuals had a *PMS2* p.Tyr191Cys variant. In addition, two individuals had a *BRCA1* p.Ala1708Val variant and another two individuals had a *FANCA* p.Cys625Ser variant. Additionally, we identified 18 unique benign (B) or likely benign variants (LB) in 10 genes that are provided in the Data Supplement.

### Association of Variant Status With Clinical Characteristics

We evaluated whether variant status was associated with important clinical characteristics (Table 4). Because of the rarity of these variants in the population, these associations did not achieve statistical significance. However, patients with P/LP variants were more likely to be diagnosed with distant-stage disease (odds ratio [OR], 3.87; 95% CI, 0.86 to 12.52) and have a higher PSA level (OR, 2.16; 95% CI, 0.95 to 4.77) than patients without identified P/LP. Additionally, carriers were not more likely to have first-degree relatives with prostate cancer (OR, 0.90; 95% CI, 0.37 to 1.97) or other cancers. When examining only carriers who had P/LP variants only in DDR genes, being diagnosed with distant-stage disease (OR, 4.30; 95% CI, 0.95 to 14.07) and having a high PSA level (OR, 2.45; 95% CI, 1.02 to 5.79) remained or became more significant (Data Supplement). Carriers were significantly more likely to have first-degree relatives with DDR gene–associated cancers (OR, 2.39; 95% CI, 1.05 to 5.77), but not exclusively with prostate cancer.

**TABLE 4.**
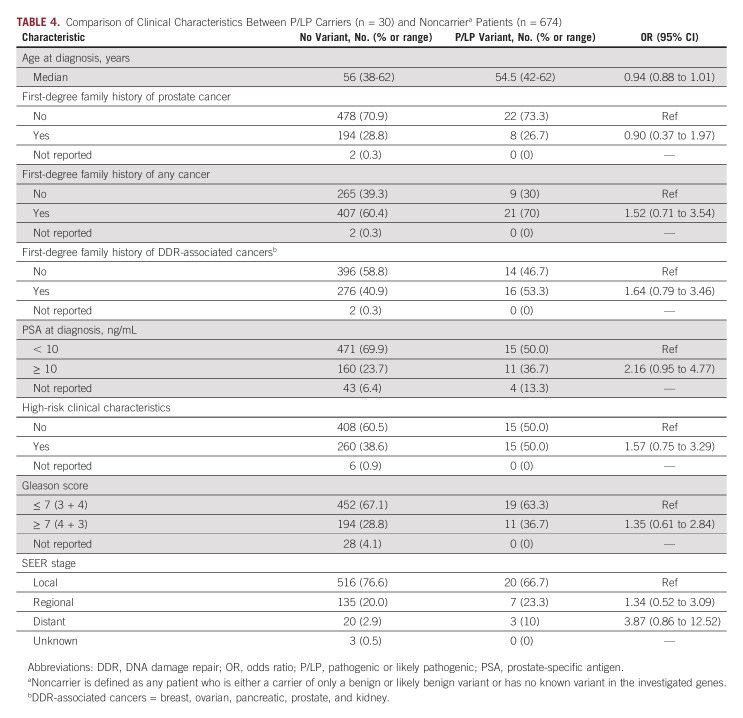
Comparison of Clinical Characteristics Between P/LP Carriers (n = 30) and Noncarrier^a^ Patients (n = 674)

We also examined the potential significance of VUS by denoting patients with such a variant as carriers, and then excluding patients who were identified to have a P/LP variant (Data Supplement). We did not find any significant differences between carriers and noncarriers of VUS variants in most clinical characteristics; however, age at diagnosis was associated with carrier status with the effect size in the opposite direction (OR, 1.1; 95% CI, 1.02 to 1.21).

## DISCUSSION

The current study advances our understanding of germline genetic variations among Black men diagnosed with early-onset prostate cancer. We identified P/LP variants in a number of DDR genes and *HOXB13*, some of which have not been previously identified in prostate cancer, further emphasizing the importance of these genes in carcinogenesis. Specifically, variants were found in *ATM*, *ATR*, *BRCA1*, *BRCA2*, *BRIP1*, *CHEK2*, *ERCC5*, *FANCA*, *HOXB13, MRE11*, *MSH6*, *PALB2*, *PMS2*, and *WRN*, largely consistent with prior published reports among individuals with metastatic or aggressive, familial, and/or early-onset prostate cancer.^12,13,22,23^ The overall prevalence of P/LP variants in our study (4.0%) is higher compared with prior reports in Black men, but lower than most estimates among non-Hispanic White men diagnosed with similar-stage disease.^24^

The prevalence of VUS in our study (5.3%) is lower compared with prior reports.^25-28^ As underscored by Kim et al,^29^ proper classification of variants, application of the ACMG/SVI guidelines, and manual review are time-consuming processes even with the help of tools like Varsome. Compared with that of the other studies, a conservative selection criterion (described in the Data Supplement) was used to identify variants of interest for the study. Nonsynonymous variants make up the majority of identified VUS in our study and others,^28^ and the stringent REVEL score threshold of 0.75, while chosen for higher specificity for disease mutations,^30^ reduces the number of variants to classify by over three-fold (data not shown). Furthermore, Black men are under-represented in studies of germline testing and have higher rates of VUS relative to White men.^25-28^ The lack of diversity in genetic ancestry within the databases and lack of follow-up of VUS impact in the literature hinder attempts to more definitively reclassify and interpret VUS. In a study of 1,051 VUS in Asians undergoing germline genetic testing over 6 years, only 8.1% (85 of 1,051) were reclassified, with 94.1% (80 of 85) being downgraded to B/LB and only 5.9% (5 of 85) reclassifications upgraded to P/LP.^31^ This suggests a bias to B/LB in identified VUS lists and may explain why, in our study, the age of diagnosis was associated with carrier status of VUS, however the effect size was in the opposite direction.

*BRCA1* and *BRCA2* have been the most widely studied genes in prostate cancer, as these tumor suppressor genes are involved in the maintenance of genomic stability through double-strand DNA repair, and variants have been linked to both early-onset and hereditary prostate cancer, as well as more aggressive clinical features, time of diagnosis, and response to therapy.^32-34^ Risk of prostate cancer among *BRCA2* carriers has been estimated to be 4.45 with an absolute risk of 27% and 60% by ages 75 and 85 years, respectively, while risk of prostate cancer among *BRCA1* carriers has been estimated to be 2.35.^35^ Pritchard et al^13^ found, in a study of men with metastatic prostate cancer, 84 germline variants in 16 genes in 82 men or 11.8% of the cohort. This was significantly higher than the variant rate in men with organ-confined disease and persons unselected for cancer from the Exome Aggregation Consortium. *BRCA2* variants were the most prevalent at 5.3%, and variants in *ATM*, *CHEK2*, *PALB2*, *BRCA1*, and *RAD51D* were also more common among men with lethal prostate cancer. There are important treatment implications for men with germline variants in *BRCA1/2*. A significant survival benefit has been demonstrated in men with castrate-resistant metastatic prostate cancer and germline variants in *BRCA1/2* when treated with the poly (ADP-ribose) polymerase inhibitor olaparib^36^; this same survival benefit has not been observed among men with variants in *ATM*.^37^ However, no difference in survival has been observed in *BRCA1/2* carriers treated with other systemic therapies (chemotherapy, abiraterone, and enzalutamide).^38,39^ The evidence to support the role of *BRCA1/2* in prostate cancer is strong enough to include genetic testing among individuals who meet National Comprehensive Cancer Network guidelines based upon reported family history and/or clinical characteristics. As with most studies focused on understanding the contribution of inherited genetic predisposition to prostate cancer, the evidence to date for the role of these DDR genes on risk and disease progression is based predominately on studies of non-Hispanic White men, making investigations like ours critical for understanding the genetic landscape in this high-risk population. As previously mentioned, in a small pilot of Black men diagnosed with early-onset disease, we discovered three protein-truncating variants in *BRCA2* and *BRIP1* as well as several private missense variants in *BRCA1/2, ATM*, and other DDR genes serving as the catalyst for the current investigation.^15^

As mentioned, the G84E variant in *HOXB13* was first discovered by our team in 2012 in a linkage study of men with hereditary and early-onset disease.^10^ This rare, moderately penetrant missense variant has since been one of most consistently replicated of all gene discoveries in prostate cancer. This variant is almost exclusively observed among men of European ancestry, with variable risk estimates.^40^ The G84E variant is more common among men with a strong family history of prostate cancer and those diagnosed at younger ages.^13^ Furthermore, Storebjerg et al^41^ demonstrated that the variant is more frequently observed in men with a higher PSA at diagnosis, higher Gleason score, and higher likelihood of positive surgical margins at the time of radical prostatectomy than noncarriers, indicating that this genetic variant may also be associated with aggressive disease. It has been demonstrated that 33%-60% of G84E carriers develop prostate cancer compared with 11.2% of the general population.^42,43^ Mechanisms underlying the increased risk of prostate cancer in patients harboring this particular variant are based on its location within the MEIS interaction domain of *HOXB13*, which regulates organ homeostasis and inhibits tumor formation.^44^ Although this variant is not commonly observed in Black men, it was discovered in three men in this study presumed to track with European ancestral portion of the genome.^10,43,45^ A recently discovered rare deletion variant in *HOXB13* (X285K), seen only among men of West African ancestry,^46-48^ was also observed in three men in our study. Its association with age at onset and advanced disease in the current study and others^47,48^ warrants further study in larger cohorts of Black men.

Interestingly, several variants were observed in genes that have not been well characterized in patients with prostate cancer, including the tumor suppressor gene *FANCA* as well as *WRN*, which is associated with the premature aging disease Werner's syndrome. *FANCA* is a noteworthy DDR gene because germline loss of function may be associated with an autosomal dominant predisposition to prostate cancer^49-51^ and it has been reported that the frequency of somatic variants in *FANCA* is increased in metastatic castrate-resistant prostate tumor tissue.^12,52^ A recent cross-sectional study of a multiracial and multi-ethnic cohort (Ashkenazi Jewish, non-Hispanic White, Black, and Hispanic) detected two *FANCA* alterations among 194 patients with prostate cancer.^53^ By comparison, the study identified no pathogenic variants in *FANCA* in a sample of 3,679 patients with no known cancer indication used as a reference group, suggesting germline *FANCA* variants may influence susceptibility to prostate cancer. Although *WRN* is typically associated with the phenotypic effects in Werner's syndrome, an analysis of a patient with a family history of prostate cancer by next-generation sequencing identified heterozygosity for the *WRN* G327X variant.^54^ The WRN protein, along with ATM, BRCA1, BRCA2, and RAD51 among others, comprises a DNA repair system by homologous recombination, indicating that its alterations may increase susceptibility to prostate cancer.

Overall genetic variation in *PMS2* may also be clinically relevant, as five unique P/LP and VUS variants were identified with four individuals. As an essential component of DNA mismatch repair, *PMS2* encodes a protein that forms a heterodimer with MLH1 and this complex interacts with MSH2 bound to mismatched bases. Importantly, defects in this gene are associated with Lynch syndrome or hereditary nonpolyposis colorectal cancer,^55^ and potentiate the formation of supratentorial primitive neuroectodermal tumors.^56^ Consequently, the potential relevance of *PMS2* genetic variation in prostate cancer development appears logical and may be worth analyzing in future sequencing studies. It is important to note that in a previous study of *PMS2* variants, carriers were at an increased risk of developing colorectal cancer compared with the general population, but there was no clear evidence of an increased risk of ovarian, gastric, hepatobiliary, bladder, renal, brain, breast, prostate, or small bowel cancer.^57^ However, this study was performed in non-Hispanic White men, and does not preclude the potential importance of this gene in Black men who develop early-onset prostate cancer. It is also plausible that the *PMS2* variants identified in this study were not detected.

Many bioinformatics pipelines do not accurately identify or classify multiple-nucleotide variants leading to incorrectly identified single-nucleotide variants (SNPs) with potential biasing results.^58^ For example, we identified a SNP in *POLE* that alone was a stop-gain loss of function variant classified as a P/LP; however, another SNP on the same allele and codon, when taken together, result in a nonsynonymous variant classified as a SNP. By actively testing for multiple nucleotide variants, we were able to provide a more accurate analysis for our study.

This study has several important strengths including its sample size, as it is one of the largest population-based investigations of early-onset prostate cancer in Black men. Despite this, the rarity of these mutations in our study population did not allow for adequate examination of individual variants and clinical characteristics. The population-based nature of the investigation increases the likelihood that the results are generalizable to the larger population of Black men diagnosed with early-onset prostate cancer. Furthermore, the identification of study participants through one of the founding members of National Cancer Institute's SEER cancer program allowed not only for the abstraction of relevant clinical data but also histopathologic confirmation of diagnosis. Finally, using a strategy that focused on a population likely to be genetically enriched increased the likelihood of detection of rare pathogenic variants. The limitations of this study include the retrospective design and lack of follow-up over time. Investigations into early-onset disease face the confounding questions as to why an individual was detected early (eg, a recent family history change increased awareness), if their cancer could have become aggressive if not caught early, or if the individual will face recurrence. The answers to these questions could affect the associations of variants with disease. Finally, the current investigation does not address the contribution of common variants on early-onset prostate cancer in Black men. Race-specific polygenic risk scores are emerging as a tool to assess cancer risk associated with common variants and should be considered in future studies in this population.

This study demonstrates the critical importance of examining under-represented patient populations for genetic risk factors related to common malignancies such as prostate cancer. Our analysis identified variants previously characterized in cohorts of non-Hispanic White men, while also characterizing several novel variants. In addition, our study indicates that carriers for P/LP variants or VUS often have a family history of cancer, and may have an increased susceptibility to developing prostate cancer at an earlier age, as well as developing aggressive disease. To build on the results of this study, future research will benefit from examining the heritability of variants in DDR genes and *HOXB13* to determine whether first-degree relatives with the same genotype truly have an increased susceptibility to developing cancer, and whether carriers are at an increased risk of developing malignancies other than prostate cancer. It is also essential to further characterize the genetic variation among Black patients with prostate cancer in separate cohorts to validate these findings, which will ultimately contribute to the understanding of a patient population. These investigations will provide insight into the unique susceptibility Black men have to developing prostate cancer, and may help reduce the health disparities these individuals face in receiving adequate health care.

## Data Availability

The data that support the findings of this study are available from the corresponding author upon reasonable request.
